# Assessing PM_2.5_ pollution in the Northeastern United States from the 2023 Canadian wildfire smoke: an episodic study integrating air quality and health impact modeling with emissions and meteorological uncertainty analysis

**DOI:** 10.1088/1748-9326/ae10c9

**Published:** 2025-10-17

**Authors:** Hao He, Timothy P Canty, Russell R Dickerson, Joel Dreessen, Amir Sapkota, Michel Boudreaux

**Affiliations:** 1 Department of Atmospheric and Oceanic Science, University of Maryland, College Park, MD 20742, United States of America; 2 Air and Radiation Administration, Maryland Department of the Environment, Baltimore, MD 21230, United States of America; 3 Department of Epidemiology and Biostatistics, School of Public Health, University of MarylandCollege Park, MD 20740, United States of America; 4 Department of Health Policy and Management, School of Public Health, University of Maryland, College Park, MD 20740, United States of America

**Keywords:** wildfire, air quality, CMAQ, BenMAP

## Abstract

Between June 6 and 8, 2023, wildfires in Quebec, Canada generated massive smoke plumes that traveled long distances and deteriorated air quality across the Northeastern United States (US). Surface daily PM_2.5_ observations exceeded 100 *µ*g m^−3^, affecting major cities such as New York City and Philadelphia, while many areas lacked PM_2.5_ monitors, making it difficult to assess local air quality conditions. To address this gap, we developed a WRF-CMAQ-BenMAP modeling system to provide rapid, spatially continuous estimates of wildfire-attributable PM_2.5_ concentrations and associated health impacts, particularly benefiting regions lacking air quality monitoring. CMAQ simulations driven by two wildfire emissions datasets and two meteorological drivers showed good agreement with PM_2.5_ observations, with linear regression results of *R^2^*∼0.6 and slope ∼0.9. We further quantified uncertainties introduced by varying emissions and meteorological drivers and found the choice of wildfire emissions dataset alone can alter PM_2.5_ simulations by up to 40 *µ*g m^−3^ (∼40%). Short-term health impacts were evaluated using the BenMAP model. Validation against asthma-associated emergency department (ED) visits in New York State confirmed the framework’s ability to replicate real-world outcomes, with ED visits increased up to ∼40%. The modeling results identified counties most severely affected by wildfire plumes, the majority of which lack regulatory air quality monitors. Our approach highlights the value of integrated modeling for identifying vulnerable populations and delivering timely health burden estimates, regardless of local monitoring availability.

## 1. Introduction

In recent years, climate change has intensified wildfire size and severity across North America [1–4], driving increased release and formation of air pollutants, including fine particulate matter with diameter less than 2.5 *µ*m (PM_2.5_) and ozone precursors such as nitrogen oxides (NO*_x_*) [5–7]. Although anthropogenic emissions have declined over the past decades, the growing frequency and intensity of wildfires have produced more frequent air quality episodes in the U.S [8–10], making wildfires an increasingly predominant influence on US air quality [11–14] and associated adverse health effects [15–18]. By 2050, wildfire-related PM_2.5_ exposure is projected to cause tens of thousands of excess deaths in the US under climate change [19].

In May 2023, Canada experienced record-breaking wildfire [20], with over 6000 outbreaks reported and total burned areas larger than the state of Connecticut, ∼1.8 × 10^4^ km^2^ (source CIFFC report, available at https://ciffc.net). Resulting smoke plumes not only degraded air quality across Canada and the U.S [21], but also reached Europe and Asia [22, 23]. From June 6–8, Canadian wildfire plumes infiltrated the Northeastern US, leading to dangerously high PM_2.5_ levels in major population centers, including New York City (NYC), Philadelphia, and Baltimore, and threatening the health of over 50 million people. During this three-day episode, daily mean PM_2.5_ concentrations reached ten times the annual average in NYC (>100 *µ*g m^−3^), triggering >30% surge in asthma-associated emergency room (ER) visits [24]. This event also coincided with a ground-level ozone exceedance above the National Ambient Air Quality Standards (NAAQS); however, the daily 8-hr maximum average ozone concentration (MDA8) of 76 ppb remained within typical summer levels for the region (figure S1, supplementary material). Quantifying more subtle wildfire impacts on ozone and assessing whether this episode qualifies as an ‘exceptional ozone event’ (potentially excludable from attainment determinations) will be addressed in a separate study.

To assess the broader air quality and associated health impacts of this unprecedented PM_2.5_ episode, we conducted a modeling study of the June 6–9 Canadian wildfire smoke event, simulating the transport and evolution of smoke plumes across the Northeastern US. This study focused on estimating PM_2.5_ associated morbidity from one specific health outcome: asthma.

## 2. Data and methods

**Figure 1.**
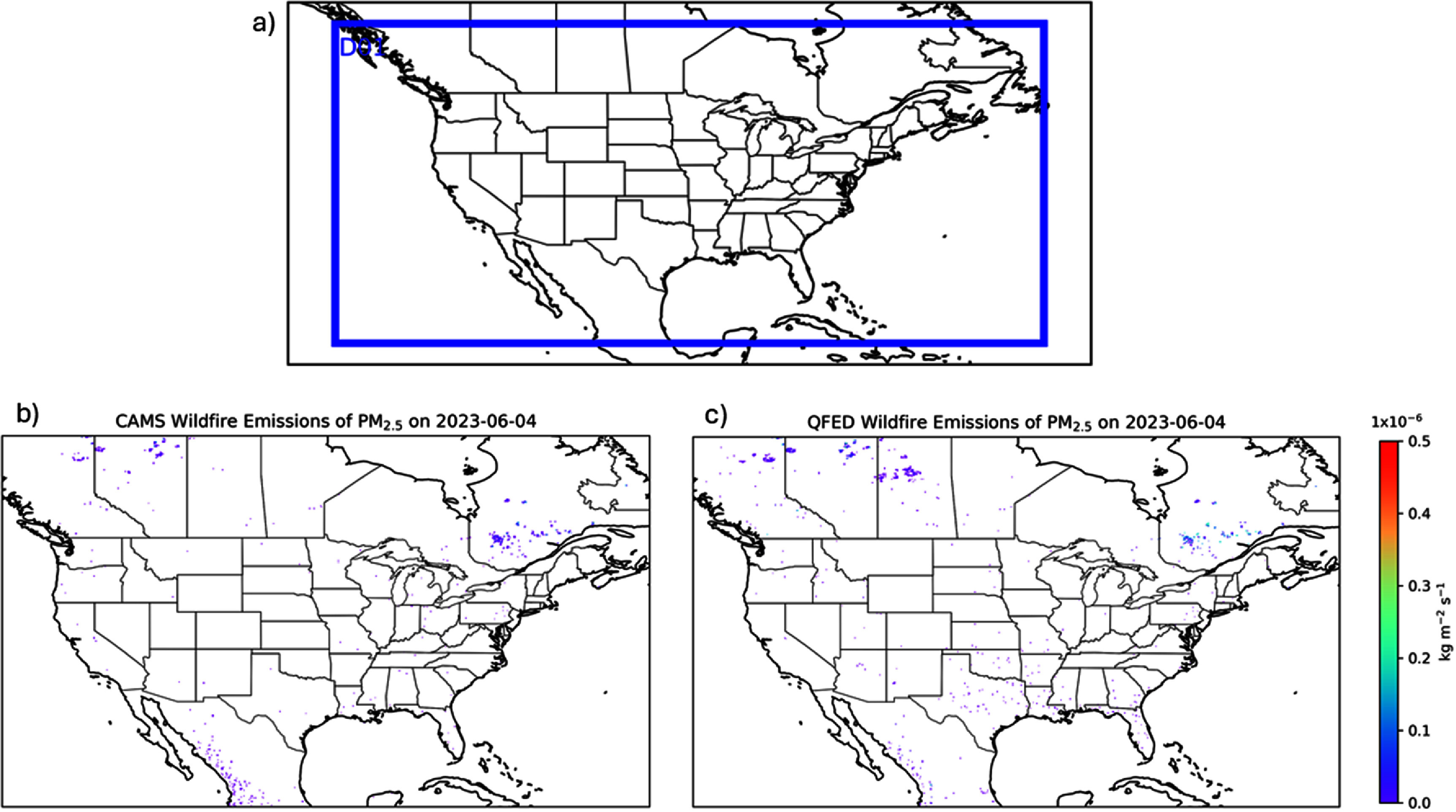
WRF-CMAQ domain and samples of satellite-based biomass burning emissions estimates. (a) 12 km CONUS domain; (b) CAMS PM_2.5_ emissions; (c) QFED PM_2.5_ emissions. Both CAMS and QFED products have 0.1° × 0.1° resolution.

We employed a regional modeling system to simulate this air quality episode, integrating the National Center for Atmospheric Research (NCAR) Weather Research & Forecasting (WRF) model version 4.5.2 [25] and the US Environmental Protection Agency (EPA) Community Multiscale Air Quality (CMAQ) model version 5.4 [26]. Simulations were conducted over the EPA 12 km Contiguous United States (CONUS) domain (12US1), which covers the wildfire sources in Quebec, Canada (figure 1(a)) and provides sufficient resolution for resolving urban areas such as NYC and facilitating county-scale health assessments. Meteorological fields were generated by WRF and processed by the Meteorology–Chemistry Interface Processor model version 5.4 following the EPA guidance [27]. To quantify the impacts of varying meteorological drivers on air quality simulations, we utilized two widely used products, the National Centers for Environmental Prediction (NCEP) Final (FNL) Operational Model Global Tropospheric Analysis and the NCEP North American Mesoscale Forecast System (NAM) datasets. We also conducted observational and analysis nudging to improve the WRF performance following the method developed in previous studies for the Northeast states [28, 29].

Anthropogenic emissions were obtained from the EPA 2016v3 platform, processed with the EPA Sparse Matrix Operator Kernel Emissions (SMOKE) model version 4.9 [30]. Biogenic emissions were calculated with the Biogenic Emissions Inventory System model version 4.0 integrated inline within CMAQ [26]. Boundary conditions and initial conditions were derived from the NCAR Whole Atmosphere Community Climate Model global simulations. Static inputs such as the ocean mask and land use land cover data were obtained from EPA.

Fire emission estimates exhibit substantial uncertainties, with regional emissions often differing by more than a factor of two across different datasets [31, 32]. To account for this variability, we selected two widely used fire emissions estimates, the European Centre for Medium-Range Weather Forecasts Copernicus Atmospheric Monitoring Service (CAMS) biomass burning emissions [33] and the National Aeronautics and Space Administration (NASA) Quick Fire Emissions Dataset (QFED) products [34]. Prior modeling studies of wildfire-related pollution typically rely on a single fire product [12, 35–37], our usage of two independent fire emissions datasets can provide a valuable range for PM_2.5_ simulations due to difference in wildfire emissions. These datasets were based on Moderate Resolution Imaging Spectroradiometer fire radiative power (FRP) products, providing global coverage at 0.1° × 0.1° resolution with emission estimates for major pollutants such as NO*_x_* and PM_2.5_. Fire emissions from the major outbreak in Quebec, Canada on 4 June 2023, show substantial discrepancies between two fire products, particularly over Saskatchewan, Canada (figures 1(b) and (c)). Differences in emissions data are expected, even well-established anthropogenic emissions can have substantial uncertainties, which may affect the accuracy of air quality modeling results [38, 39]. These pronounced variations underscore the necessity of employing multiple fire datasets to capture the potential range of uncertainty in emission estimates.

CMAQ relies on fire emissions data prepared by the US EPA, typically derived from national fire information databases such as the Geospatial Multi-Agency Coordination wildland fire dataset (www.frames.gov/catalog/948) and modeling estimates such as the EPA BlueSky Smoke Modeling Framework [40, 41]. This process often results in a data availability lag of 2–3 years. By using these two near real-time global fire products, we can more promptly simulate wildfire-related air quality episodes in the United States. Since these fire products were originally developed for global chemical transport models, we speciated CAMS and QFED emissions for the Carbon Bond 6 Mechanism Revision 5 (CB6r5) [42] in CMAQ, e.g. summing pentene, hexene, octene, and higher alkenes from CAMS VOCs to the internal alkene bond variable for CMAQ. We then regridded emission onto the 12 km CMAQ grid and allocated the emissions aloft based on the plume top and bottom height products in CAMS data. Lastly, the daily CAMS and QFED data were downscaled to create hourly inputs for CMAQ.

The WRF-CMAQ system was run from May 1 to June 15, with the first two weeks as spin-up. Using two meteorological drivers and two fire emissions datasets, we conducted six CMAQ simulations: two control runs that included only anthropogenic emissions and excluded fire emissions (hereafter referred to as CMAQ-noWildfire-FNL and CMAQ-noWildfire-NAM) and four sensitivity runs that incorporated fire emissions driven by two meteorological drivers and two fire emissions datasets, referred to as CMAQ-CAMS-FNL, CMAQ-CAMS-NAM, CMAQ-QFED-FNL, and CMAQ-QFED-NAM.

CMAQ performance was evaluated against daily PM_2.5_ observations from the EPA Air Quality System (AQS) network in the Northeastern US. Health impacts from wildfire plumes were estimated with the EPA Environmental Benefits Mapping and Analysis Program—Community Edition (BenMAP-CE) model version 1.5.8 [43] driven by CMAQ outputs. While most CMAQ-BenMAP studies typically generate health assessments without additional evaluation or validation [35, 44, 45], we strengthened our analysis by gathering asthma-associated emergency department (ED) visit data in New York from two independent publications [24, 46] to validate the accuracy of BenMAP results.

## 3. Results

### 3.1. Evaluation of CMAQ simulations and uncertainty analysis

**Figure 2.**
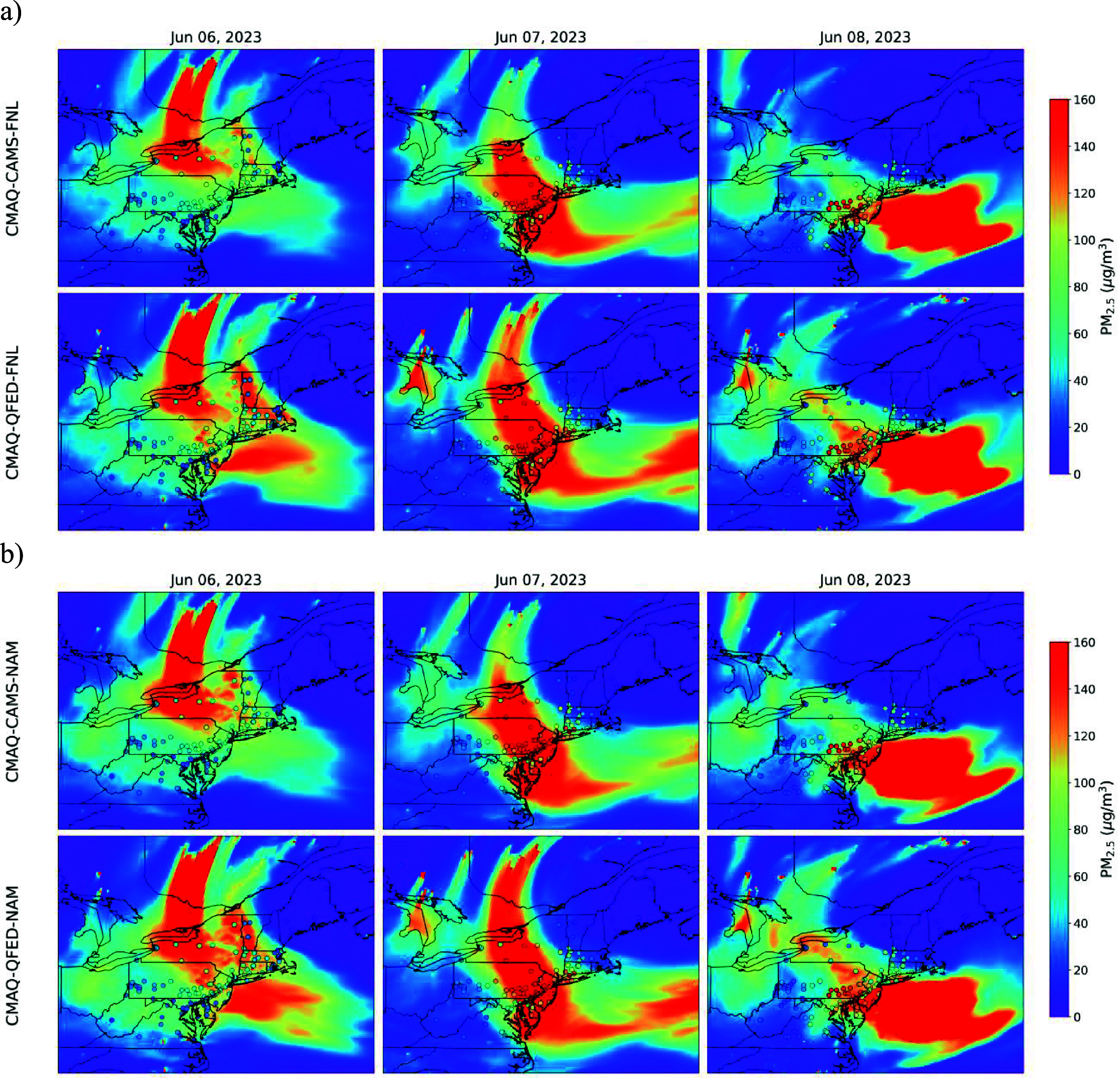
Comparison of daily PM_2.5_ concentrations from the EPA AQS sites and CMAQ simulations between 6–8 June 2023 over the Northeastern US. The filled dots represent daily PM_2.5_ levels at AQS monitoring sites; the contours represent the model simulated values. (a) and (b) Shows results from CMAQ runs driven by the FNL and NAM meteorology, respectively. In each figure, the upper/lower panel shows results from CAMS/QFED wildfire emissions, respectively. Similar plots for CMAQ runs without wildfire emissions are presented in figure S2 of the supplementary material.

**Figure 3.**
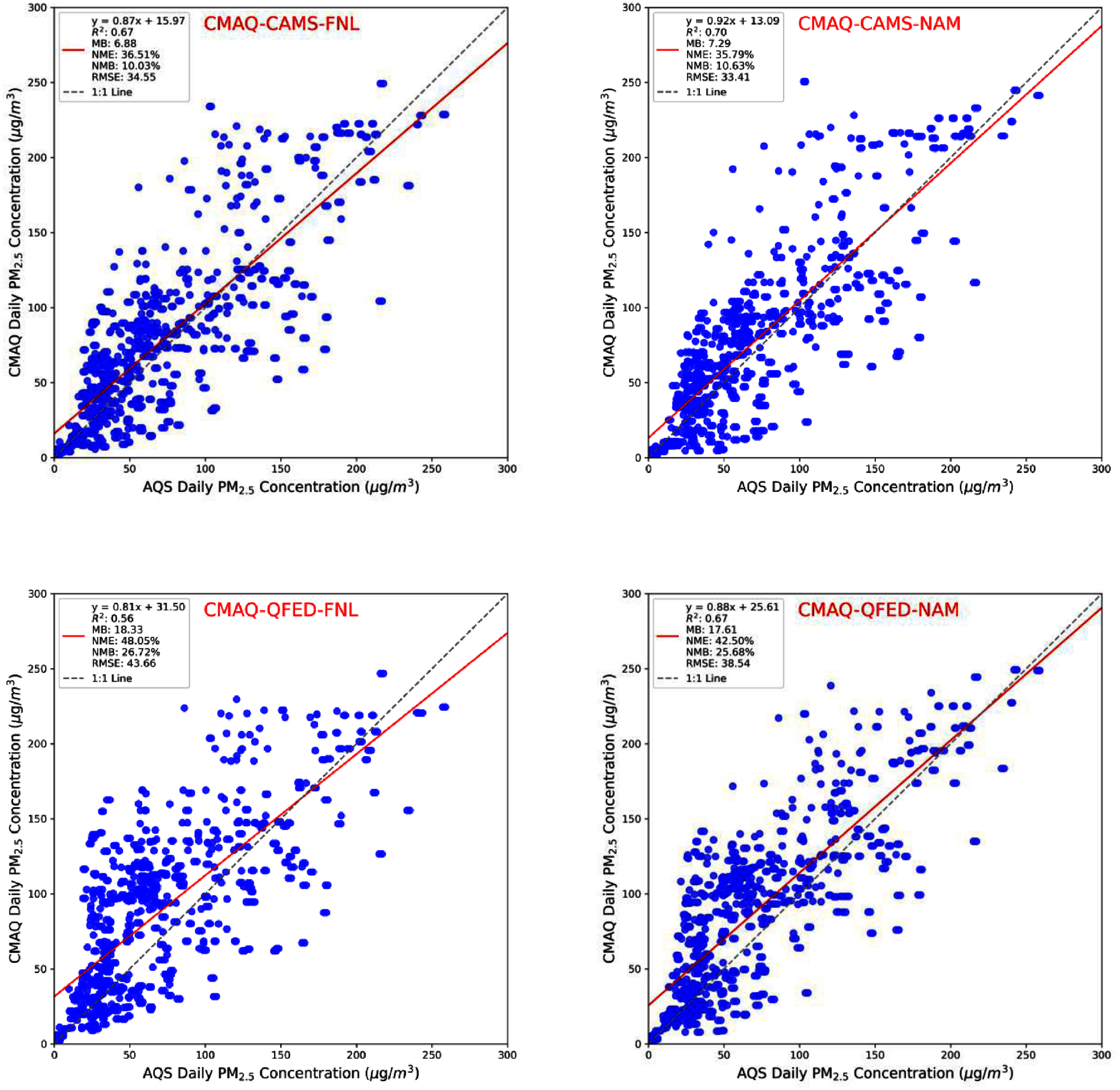
Comparison of daily PM_2.5_ concentrations from the EPA AQS sites and CMAQ simulations with wildfire emissions between 6–8 June 2023 over the Northeastern states. Similar scatter plots for CMAQ runs without wildfire emissions are presented in figure S3 of the supplementary material.

All four CMAQ simulations with wildfire emissions reproduced the 7–9 June Canadian wildfire plume transport, with only minor differences in PM_2.5_ magnitude and spatial distribution (figure 2). Linear regression analyses against EPA AQS observations showed strong correlations (*R*^2^ ∼ 0.6) and slopes close to 0.8 during this three-day episode (figure 3), suggesting the model effectively captured the regional PM_2.5_ episode. Table 1 summarizes the statistical metrics, showing that the CMAQ-CAMS-NAM run may offer the best performance, with a slope of 0.92, *R*^2^ of 0.7, mean bias (MB) of 7.29 *μ*g m^−3^, and root mean square error (RMSE) of 33.41 *μ*g m^−3^. Additionally, our results suggest that the elevated PM_2.5_ levels were not likely driven by meteorological conditions, as the two CMAQ-noWildfire runs produced extremely low PM_2.5_ concentrations compared to AQS observations.

**Table 1.** Summary of analysis of PM_2.5_ observations and CMAQ simulations for the 6–9 June 2023 episode over the AQS sites in the northeastern US. MB, NME, NMB, and RMSE represent mean bias, normalized mean error, normalized mean bias, and root mean square error. Units are *µ*g m^−3^ if not noted.

CMAQ Runs	Slope	*R* ^2^	MB	NME	NMB	RMSE
CAMS-FNL	0.87	0.67	6.88	36.51%	10.03%	34.55
CAMS-NAM	0.92	0.7	7.29	35.79%	10.63%	33.41
QFED-FNL	0.81	0.56	18.33	48.05%	26.72%	43.66
QFED-NAM	0.88	0.67	17.61	42.50%	25.68%	38.54
CMAQ-Average	0.87	0.68	12.53	38.84%	18.26%	35.43
noWildfire-FNL	0.01	0.02	−64.45	94.02%	−93.96%	83.79
noWildfire-NAM	0.00	0.01	−65.09	94.96%	−94.91%	84.39
noWildfire-Avg	0.00	0.01	−64.77	94.49%	−94.44%	84.09

To quantify the uncertainty associated with different wildfire emission datasets and meteorological drivers, we calculated the average of the four sensitivity runs that include wildfire emissions, referred to as the CMAQ-Average case, and the average of two control runs that excluded wildfire emissions, referred to as the CMAQ-noWildfires-Average case. We consider CMAQ-Average results as the best estimate (or ‘true’) CMAQ results and use it as the baseline to quantify the uncertainties associated with wildfire emissions and meteorological drivers. The CMAQ-noWildfire-Average case serves as the control run to estimate the impacts of simulated Canadian wildfire plumes on regional air quality and public health. The uncertainties associated with the wildfire emissions inputs and meteorological drivers are calculated as:

\begin{align*} {\text{emi}}{{\text{s}}_{{\text{uncertainty}}}} &amp; = \frac{1}{2} \times \left[ \left( {{\text{QFE}}{{\text{D}}_{{\text{FNL}}}} - {\text{CAM}}{{\text{S}}_{{\text{FNL}}}}} \right) \right.\nonumber\\ &amp; \quad\left. + \left( {{\text{QFE}}{{\text{D}}_{{\text{NAM}}}} - {\text{CAM}}{{\text{S}}_{{\text{NAM}}}}} \right) \right] \\ {\text{meteorolog}}{{\text{y}}_{{\text{uncertainty}}}} &amp; = \frac{1}{2} \times \left[ \left( {{\text{CAM}}{{\text{S}}_{{\text{FNL}}}} - {\text{CAM}}{{\text{S}}_{{\text{NAM}}}}} \right)\right. \nonumber\\ &amp; \quad\left. + \left( {{\text{QFE}}{{\text{D}}_{{\text{FNL}}}} - {\text{QFE}}{{\text{D}}_{{\text{NAM}}}}} \right) \right] .\end{align*}

when switching fire emissions from CAMS to QFED and meteorological inputs from NAM to FNL, respectively.

**Figure 4.**
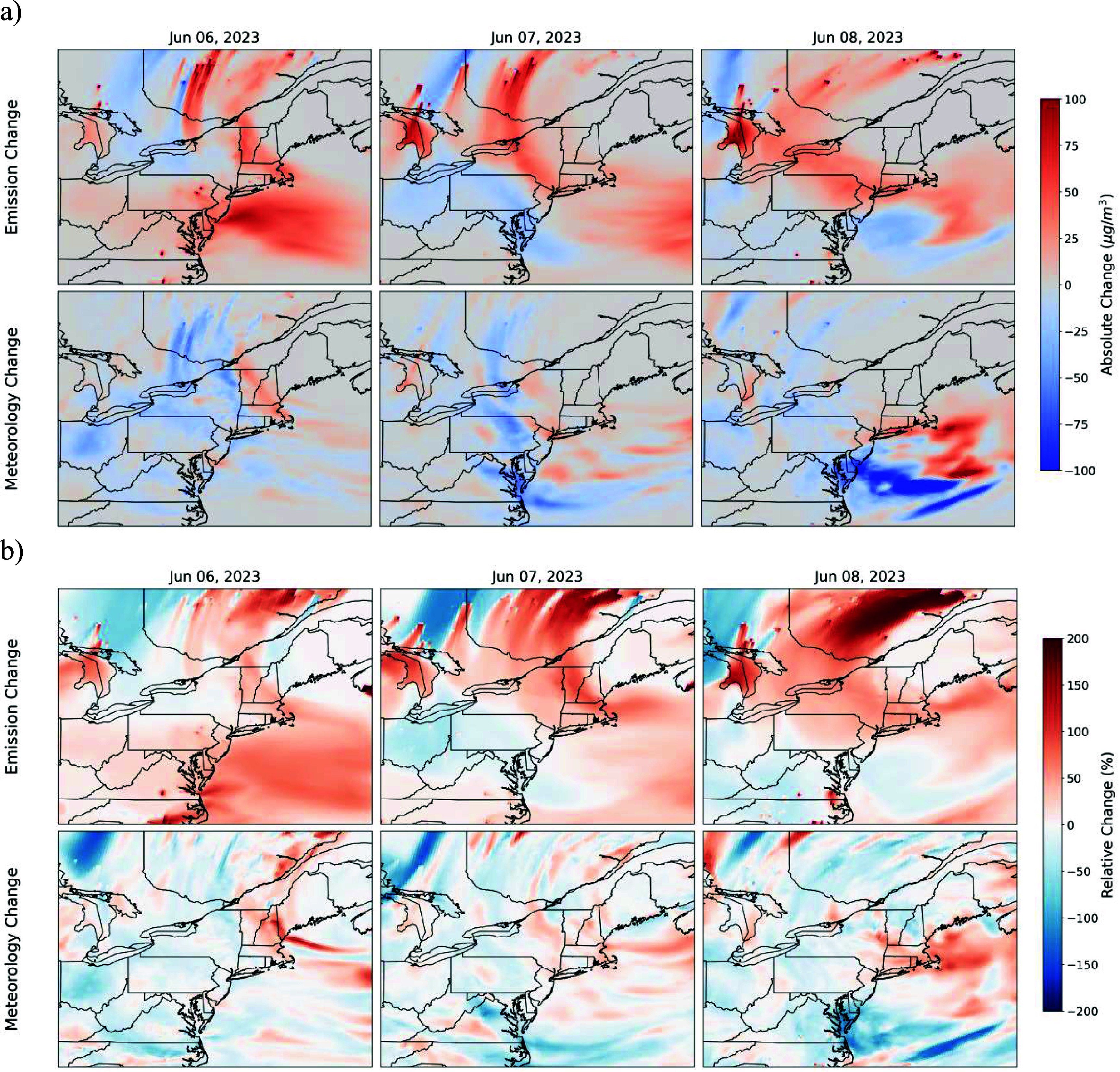
Uncertainties of wildfire emissions input and meteorological fields on the surface PM_2.5_ simulations during the 6–8 June 2023 Canadian wildfire episode over the Northeastern US. (a) absolute change when switching wildfire emissions from CAMS to QFED and switching meteorology from NAM to FNL (unit: *µ*g m^−3^); (b) relative change with respect to the CMAQ-average results.

Figure 4 presents both the absolute and relative changes in uncertainty, using CMAQ-Average as the baseline. Overall, emissions-related uncertainties are substantial; ±25 *μ*g m^−3^ (±50%) over the Northeastern US and up to up to 100 *μ*g m^−3^ (200%) over wildfire sources regions in Canada, confirming large differences in the location and intensity between satellite-based wildfire products (figure 1) that can significantly influence the simulated plume concentrations and transport. In contrast, meteorology-related uncertainties are smaller, primarily driven by variations in air mass pathways between two WRF simulations. Both uncertainties are regionally dependent, and CMAQ runs could exhibit large discrepancy at specific locations (as discussed below), while still achieving good model performance across the broader study domain (figure 3).

**Figure 5.**
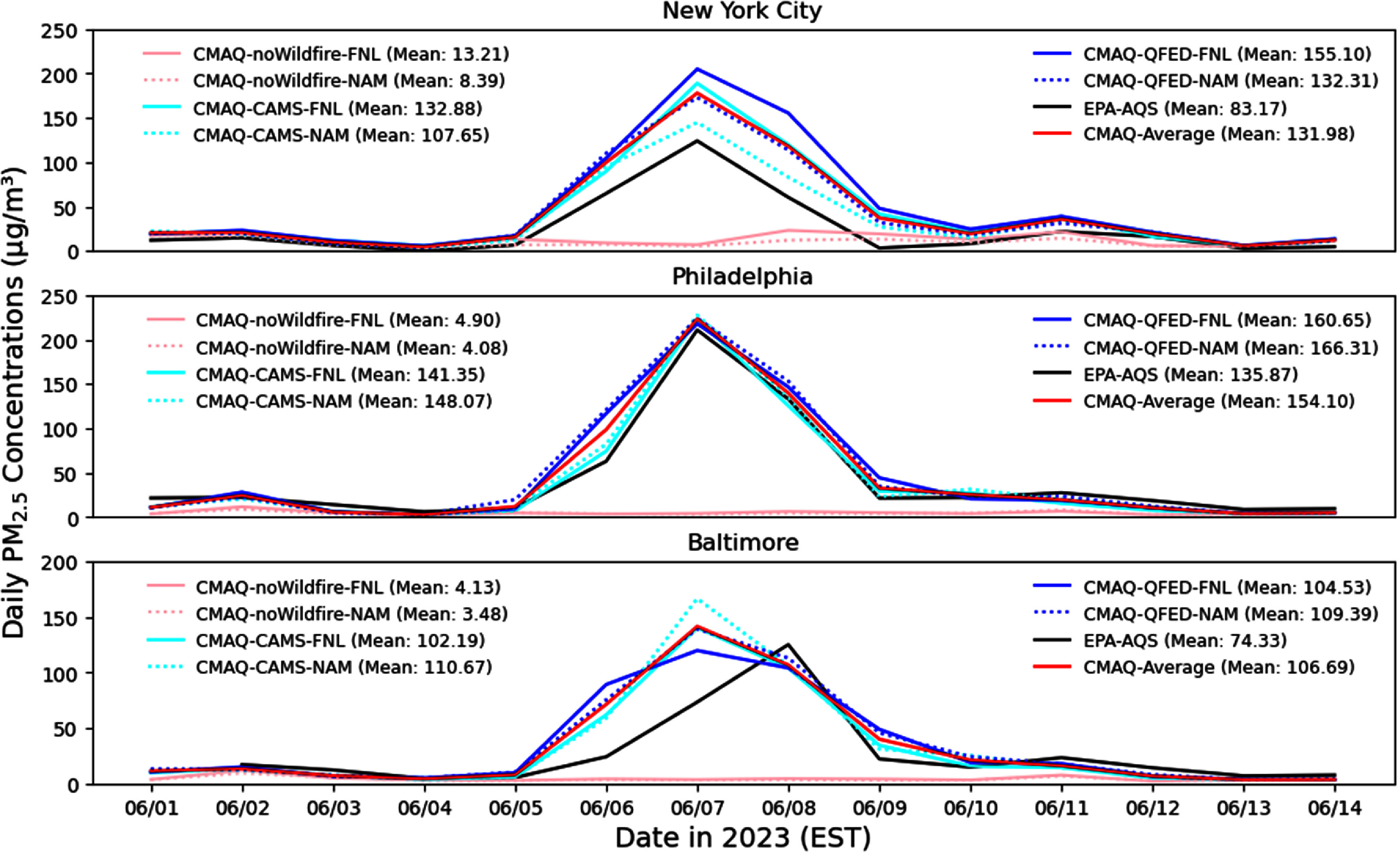
Comparison of daily PM_2.5_ concentrations from EPA AQS observations and CMAQ simulations in NYC, Philadelphia, and Baltimore in June 2023. The three-day average (6 June–8 June) of PM_2.5_ concentrations are calculated and listed in the legend. The AQS stations selected are, the City College of New York (CCNY) site (ID: 360610135, location: 40.8198° N and 73.9483° W) for NYC, the North East Waste site (ID: 421010048, location: 39.9914° N and 75.0808° W) for Philadelphia, and the Edgewood site (ID: 240251001, location: 39.4102° N, and 76.2969° W) for Baltimore.

For major cities, CMAQ well reproduced the high PM_2.5_ episode in Philadelphia and slightly overestimated the levels in NYC and Baltimore (figure 5). The PM_2.5_ peaks on 7 June in NYC and Philadelphia (124.1 *µ*g m^−3^ and 211.2 *µ*g m^−3^, respectively) were reproduced. CMAQ miss-timed the PM_2.5_ peak on 8 June in Baltimore (125.3 *µ*g m^−3^) and generated PM_2.5_ levels >100 *µ*g m^−3^ on 7 June, maybe because Baltimore is on the boundary of the wildfire plume suggested by all four CMAQ runs on 7 June (figure 2) where the exact plume location and arrival time (figure S4, supplementary material) are difficult to simulate. Conversely, CMAQ simulations without fire emissions indicated low PM_2.5_ concentrations (generally <10 *µ*g m^−3^), confirming the minimal contribution of anthropogenic sources to this episode and highlighting the dominant role of wildfire.

Table 2 summarizes the uncertainties associated with switching wildfire emissions datasets (CAMS to QFED) and meteorological drivers (NAM to FNL) over the major cities. These uncertainties show significant day-to-day and location-specific variations. The direction (sign) of the uncertainties depends on the arbitrarily defined order of comparison (i.e. CMAS to QFED); reversing the order would reverse the sign. In general, uncertainties resulting from different wildfire emissions are substantial, typically 10–40 *µ*g m^−3^ (15%–40%). In contrast, uncertainties from changing meteorological drivers are smaller, usually 5–10 *µ*g m^−3^ (5%–10%). However, notable exceptions occur on June 7 and 8 over NYC, where meteorological uncertainties exceed 40 *µ*g m^−3^ (20%–30%). Both figure 5 and table 2 demonstrate that the choice of wildfire emissions dataset and meteorological driver can introduce considerable uncertainty into PM_2.5_ simulations at specific locations and times. Nevertheless, CMAQ consistently captures the broader regional signal of the 2023 Canadian wildfire event.

**Table 2.** Uncertainty analysis from wildfire emissions and meteorological drivers for the June 2023 Canadian wildfire event. The uncertainty of wildfire emissions dataset is estimated when switching CAMS to QFED; the uncertainty of meteorological driver is estimated when switching NAM to FNL. Units are *µ*g m^−3^ and % for the relative change values in parenthesis.

		6 June	7 June	8 June
NYC	CMAQ-Average	100.1	178.2	118.0
	Emis. Uncertainty	15.9 (15.9)	23.4 (13.2)	34.0 (28.8)
	Met. Uncertainty	−4.9 (−4.9)	37.8 (21.2)	39.0 (33.0)

Philadelphia	CMAQ-Average	99.3	223.2	140.0
	Emis. Uncertainty	40.7 (41.0)	−2.4 (−1.1)	21.1 (15.1)
	Met. Uncertainty	−5.9 (−6.0)	−5.0 (−2.3)	−7.1 (−5.1)

Baltimore	CMAQ-Average	72.1	141.8	107.1
	Emis. Uncertainty	22.0 (30.4)	−22.8 (−16.1)	4.6 (4.3)
	Met. Uncertainty	8.3 (11.6)	−22.5 (−15.9)	−5.6 (−5.2)

### 3.2. Health assessment using BenMAP

**Figure 6.**
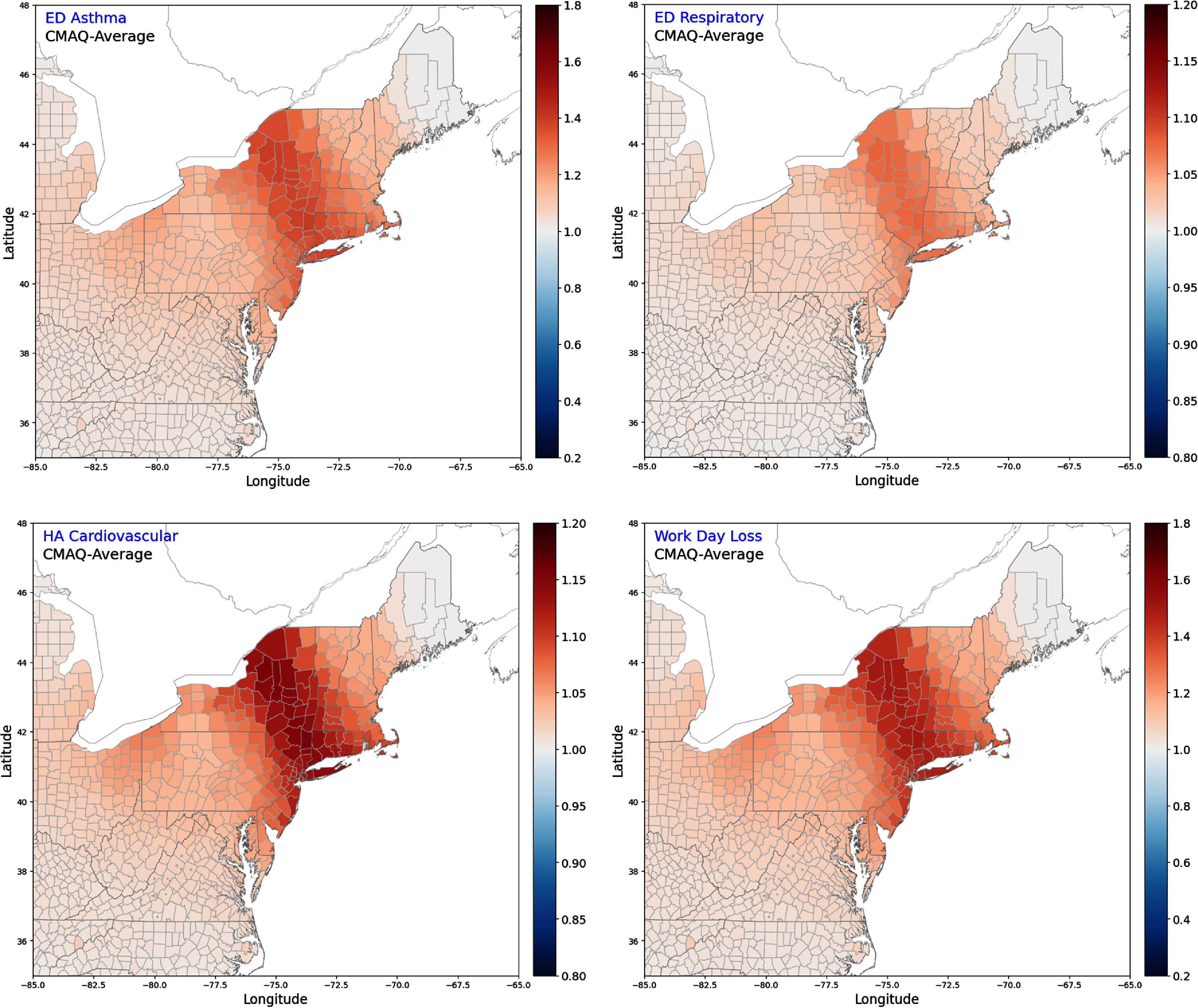
IRR values for health impacts in the Northeastern states during the Canada wildfire episode in 6–8 June 2023. BenMAP results driven by the CMAQ-average results are shown here. Similar results for four CMAQ sensitivity runs are included in figures S6–S9 of the supplementary material. (a) Asthma ED visits; (b) respiratory ED visits; (c) Cardiovascular HA; (d) work day loss.

The good model performance for the Canadian wildfire plumes over the Northeastern region supports the usage of CMAQ results to estimate health impacts. As an example, we calculated the 3 day average PM_2.5_ concentrations from six CMAQ runs, i.e. noWildFire-FNL and noWildfire-NAM (as the baseline), CAMS-NFL, CMAS-NAM, and QFED-FNL, and QFED-NAM, over the 12-km CMAQ domain, and generated air quality inputs for the BenMAP model. To determine health impacts, we selected the national 2023 population data and a health impact function based on a short-term exposure study for asthma ED visits [47], both available in BenMAP. We then calculated the incidence rate ratio (IRR) of asthma ED visits, representing the relative change in ED visits due to changes in ambient air quality, at the county level for the Northeast US (figure 6(a)). The four CMAQ runs driven by different wildfire emissions and meteorological fields produced consistent IRR estimates by BenMAP, supporting the credibility of our modeling system.

For model validation of BenMAP, we used asthma ED visit data from two publications [24, 46] and calculated IRR values for five boroughs in NYC and three regions in New York State (figure S5, supplementary material). Following Chen *et al* [24], we defined asthma ED visit numbers before and after the three-day episode as the baseline (non-wildfire) period and the data during June 6–8 as the smoke wave. IRR values were calculated based on data from Meek [46] and BenMAP simulated numbers for June 2023. The BenMAP modeled IRR is calculated as:

\begin{equation*}{\text{IRR}} = \,\frac{{\mathop \sum \nolimits \Delta {\text{Health}}\,{\text{Events}}}}{{\mathop \sum \nolimits {\text{Baseline}}\,{\text{Events}}}} + 1\end{equation*}

where ‘ΔHealth Events*’* refers to the best estimate of a health impact due to a change in air pollution and ‘Baseline Events’ is the baseline scenario before the change. The change in health outcomes is calculated using BenMAP’s epidemiology health impact functions:

\begin{equation*}\Delta Y = {y_0} \times {\text{Pop}} \times \left( {1 - {{\text{e}}^{ - \beta \Delta x}}} \right)\end{equation*}

where Δ*Y* is the estimated change in health outcome, *y_0_* is the baseline incidence rate, Pop is the exposed population, Δ*x* is the change in pollution concentration, and *β* is the concentration-response coefficient. BenMAP reports estimate of Health Events and their associated variance (standard deviation) at the county level. Assuming that the BenMAP health residuals have constant variance and follow a normal distribution, 95% confidence interval (CI) of the total change can be estimated as

\begin{align*} {\text{C}}{\text{I}_{95\% }} &amp; = \mathop \sum \nolimits \Delta {\text{Health}}\,{\text{Events}} \pm 1.96 \nonumber\\ &amp; \quad \times \sqrt {\mathop \sum \nolimits {\text{Variance}}} \hfill \\ {\text{IR}}{\text{R}_{95\% {\text{CI}}}} &amp; = \left[ \frac{{\text{C}{\text{I}_{95\% {\text{Lower}}}}}}{{\mathop \sum \nolimits {\text{Baseline}}\,{\text{Events}}}} \right.\nonumber\\ &amp; \quad\left. + 1,\,\frac{{\text{C}{\text{I}_{95\% {\text{Upper}}}}}}{{\mathop \sum \nolimits {\text{Baseline}}\,{\text{Events}}}} + 1 \right] .\end{align*}

**Figure 7.**
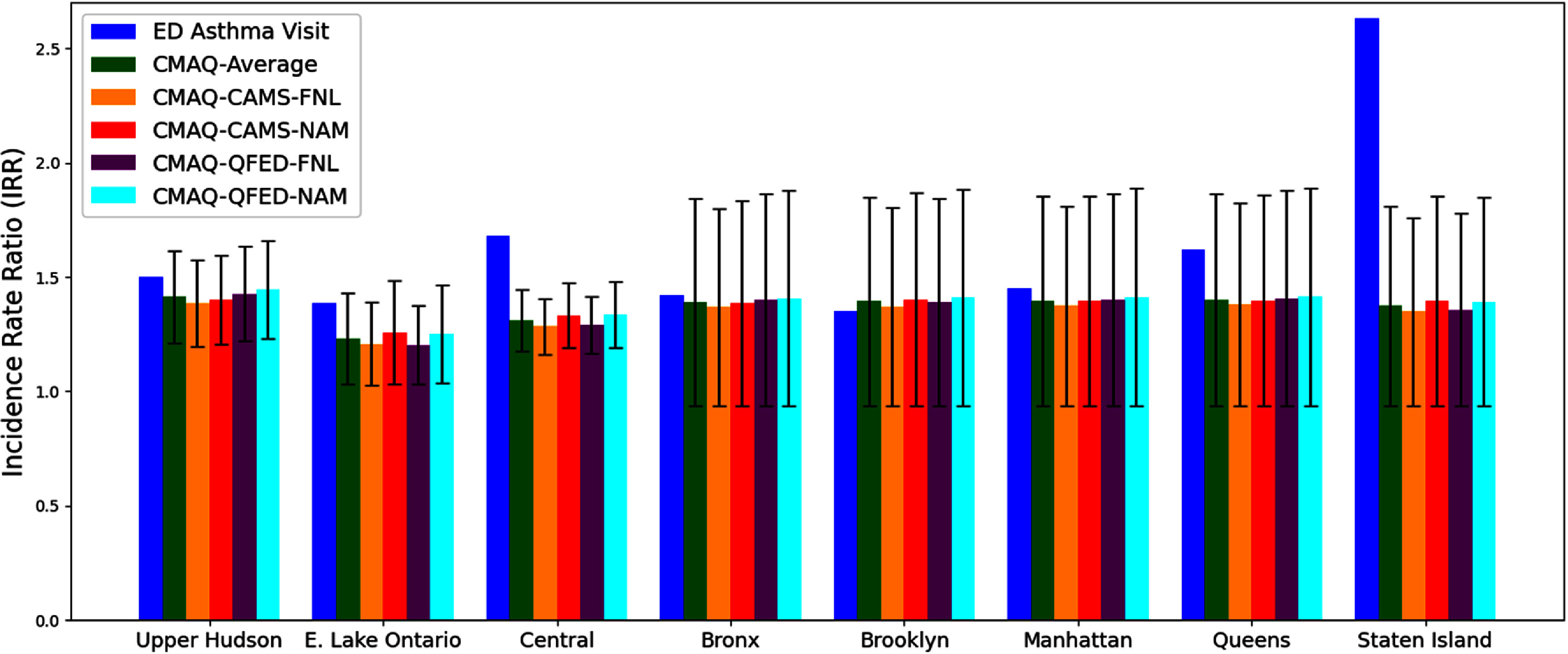
IRR values with 95% CI for asthma ED visit number and BenMAP results during the Canadian wildfire episode in 6–8 June 2023. The black error bars represent the range of lower and upper bounds of 95% CI of IRR. Detailed values are listed in table S1 of the supplementary material. NYC includes five boroughs: Bronx, Brooklyn, Manhattan, Queens, and Staten Island. The Upper Hudson region includes Albany, Columbia, Fulton, Greene, Montgomery, Rensselaer, Saratoga, Schenectady, Schoharie, and Washington counties; the Eastern Lake Ontario region includes Jefferson, Monroe, Oswego, and Wayne counties; the Central region includes Allegany, Broome, Cayuga, Chemung, Chenango, Cortland, Delaware, Herkimer, Livingston, Madison, Oneida, Onondaga, Ontario, Otsego, Schuyler, Seneca, Steuben, Tioga, Tompkins, and Yates counties.

Figure 7 and table S1 in the supplementary material shows that BenMAP reasonably captured changes in asthma ED visits with most results falling with 95% CI, aligning well with changes calculated based on administrative records of ER visits for the Upper Hudson region, Eastern Lake Ontario region, Central New York, and four boroughs in NYC. However, a substantial underestimation was observed for Staten Island, which has a significantly higher IRR value compared to other boroughs in NYC [24].

Figure 6(a) highlights the counties with largest IRR values for asthma ED visits in the Northeastern United States. Table 3 lists the top 15 counties with the highest IRR values across the four CMAQ simulations with wildfire emissions, with 9 out of 15 counties appearing on all the lists. These counties exhibit IRR values of ∼1.40, equivalent to a 40% increase in asthma ED visits during the wildfire episode. Notably, most of these high-risk counties (9/14/9/11 out of 15 in the CAMS-FNL/CAMS-NAM/QFED-FNL/QFED-NAM, respectively) lack EPA AQS monitoring sites, limiting ‘real-time’ air quality tracking and timely public health advisories. This monitoring gap might increase risks for vulnerable populations, including children and individuals with pre-existing respiratory conditions like asthma, during hazardous air quality episodes.

**Table 3.** Lists of top 15 counties with the highest IRR values for asthma ED visits, as calculated in BenMAP based on CMAQ PM_2.5_ concentrations. Column FIPS: Federal information processing standards (FIPS) code; Column AQS: ‘Yes/No’ indicates whether an EPA AQS station is in the county. State and County names in italics indicate counties that appear on all the lists. NY: New York State; CT: state of Connecticut.

CMAQ-CAMS-NAM						CMAQ-QFED-NAM				
FIPS	IRR	County	State	AQS		FIPS	IRR	County	State	AQS

36043	1.41	*Herkimer*	*NY*	No		36027	1.43	*Dutchess*	*NY*	No
36095	1.40	Schoharie	NY	No		36111	1.42	*Ulster*	*NY*	No
36049	1.40	*Lewis*	*NY*	No		36049	1.42	*Lewis*	*NY*	No
36065	1.39	Oneida	NY	Yes		36043	1.42	*Herkimer*	*NY*	No
36111	1.39	*Ulster*	*NY*	No		36039	1.42	Greene	NY	No
36027	1.39	*Dutchess*	*NY*	No		36079	1.41	*Putnam*	*NY*	No
36057	1.39	*Montgomery*	*NY*	No		36057	1.41	*Montgomery*	*NY*	No
36035	1.39	*Fulton*	*NY*	No		36041	1.41	*Hamilton*	*NY*	No
36077	1.38	Otsego	NY	No		36035	1.41	*Fulton*	*NY*	No
36041	1.38	*Hamilton*	*NY*	No		09001	1.41	*Fairfield*	*CT*	Yes
36025	1.38	Delaware	NY	No		36021	1.41	*Columbia*	*NY*	No
36079	1.38	*Putnam*	*NY*	No		36071	1.40	Orange	NY	Yes
09001	1.37	*Fairfield*	*CT*	Yes		36065	1.40	Oneida	NY	Yes
36021	1.37	*Columbia*	*NY*	No	09005	1.40	Litchfield	CT	Yes

**CMAQ-CAMS-FNL**						**CMAQ-QFED-NFL**				
FIPS	IRR	County	State	AQS		FIPS	IRR	County	State	AQS

*36049*	*1.37*	*Lewis*	*NY*	*No*		*36027*	*1.41*	*Dutchess*	*NY*	*No*
*36043*	*1.37*	*Herkimer*	*NY*	*No*		*36049*	*1.40*	*Lewis*	*NY*	*No*
*36027*	*1.37*	*Dutchess*	*NY*	*No*		*36035*	*1.40*	*Fulton*	*NY*	*No*
*36035*	*1.37*	*Fulton*	*NY*	*No*		09005	1.39	Litchfield	CT	Yes
*36057*	*1.37*	*Montgomery*	*NY*	*No*		*09001*	*1.39*	*Fairfield*	*CT*	*Yes*
36039	1.36	Greene	NY	No		*36043*	*1.39*	*Herkimer*	*NY*	*No*
*36041*	*1.36*	*Hamilton*	*NY*	*No*		*36057*	*1.39*	*Montgomery*	*NY*	*No*
36065	1.36	Oneida	NY	Yes		*36041*	*1.39*	*Hamilton*	*NY*	*No*
36095	1.36	Schoharie	NY	No		36103	1.39	Suffolk	NY	Yes
*09001*	*1.36*	*Fairfield*	*CT*	*Yes*		*36111*	*1.39*	*Ulster*	*NY*	*No*
*36111*	*1.36*	*Ulster*	*NY*	*No*		36021	1.39	Columbia	NY	No
36021	1.36	Columbia	NY	No		36039	1.39	Greene	NY	No
36103	1.36	Suffolk	NY	Yes		36071	1.39	Orange	NY	Yes
9005	1.35	Litchfield	CT	Yes		*36079*	*1.39*	*Putnam*	*NY*	*No*
*36079*	*1.35*	*Putnam*	*NY*	*No*	9009	1.38	Newe Haven	CT	Yes

The strong agreement between BenMAP-estimated asthma ED visits and published observations support the CMAQ-BenMAP framework as a reliable tool for assessing additional health outcomes. Accordingly, we extended the analysis using three additional short-term PM_2.5_ exposure epidemiological health impact functions for respiratory disease ED visits [48], cardiovascular disease hospital admissions (HA) [49], and work loss days [50]. Figures S6–S9 in the supplementary material show that all these three health impacts exhibit spatial patterns similar to those for asthma ED visits (figures 6(b)–(d)), with estimated increase of up to ∼8% for respiratory disease ED visits, ∼15% for cardiovascular HA, and ∼50% for work loss days, all attributable to wildfire-related PM_2.5_ exposure.

## 4. Conclusions and discussion

We developed a regional modeling system to simulate the June 2023 Canadian wildfire events, that significantly impacted air quality over the Northeastern US Driven by two biomass burning emissions datasets (CAMS and QFED) and two meteorological drivers (FNL and NAM), the system effectively captured wildfire plume transport and associated PM_2.5_ pollution, demonstrating good agreement with EPA AQS observations. These results confirm that elevated surface PM_2.5_ levels were primarily attributable to Canadian wildfire plumes, underscoring their dominance of air pollution on these days. Our uncertainty analysis further indicates that the choice of wildfire emissions datasets exerts a greater impact on simulated PM_2.5_ levels, compared to the impacts from different meteorological drivers.

To assess the public health implications, we utilized the BenMAP model driven by CMAQ, estimating wildfire-associated health burdens at the county scale across the Northeastern US. These BenMAP results align well with observed asthma ED visits in New York State, with values falling within 95% CI. In the most impacted counties, IRR values for asthma ED visits reached up to 1.40, indicating a 40% increase. These findings underscore the severe public health risks posed by wildfire-related air pollution and highlight the capability of a regional modeling system to provide seamless estimates of both air pollution and associated health impacts. Future improvements should refine emission inventories and enhance model representations of wildfire smoke and interactions with atmospheric processes.

We observed a discrepancy between the highest IRR values in New York State (figure 6) and the highest simulated PM_2.5_ concentrations in eastern Pennsylvania (figure 2). This suggests that wildfire PM_2.5_ may have heterogeneous impacts across asthma-affected populations in BenMAP, which estimates health impacts using baseline incidence rates derived from the Healthcare Cost and Utilization Project data [51], including state-reported datasets such as the State Inpatient Databases and the State Emergency Department Databases [52]. County-specific baseline rates in BenMAP may be higher in New York than in Pennsylvania, leading to greater estimated health impacts despite similar or lower PM_2.5_ levels. While identifying the key moderating factors is beyond the scope of this study, the health effects of smoke events may vary due to factors such as a community’s prior exposure to wildfire smoke, a potential issue that warrants further investigation in future.

Despite providing valuable insights, our modeling framework has limitations. CMAQ underestimated PM_2.5_ in NYC and Baltimore compared to Philadelphia. As shown in figures 2–5, both cities sit on the periphery of the simulated plume, where PM_2.5_ concentration gradients were particularly steep, due to the uncertainties from varying wildfire emissions and meteorological drivers, a challenging feature for numerical models to capture accurately. Key sources of these biases include: (1) imperfect meteorology, which affects plume transport even when multiple meteorological datasets were used; (2) uncertainties in fire emissions datasets, arising from FRP detection methods, emissions factors determination, and plume rise algorithms. Our study employed six computationally efficient sensitivity experiments; future work should adopt more rigorous approaches, such as ensemble meteorological simulations and optimized fire emissions, to reduce uncertainty.

Limitations also exist in the BenMAP health assessment. BenMAP applies national baseline incidence rates, which may overlook local variability in population vulnerability; it accounts only for PM_2.5_, ignoring specific wildfire chemical species, and its short-term exposure health functions are based on typical ambient PM_2.5_ levels, introducing uncertainty when extrapolated to high concentrations during this episode. In addition, because 2023 hospitalization records for our study area were unavailable, we relied on BenMAP built-in datasets to estimate potential health impacts. Future work should directly quantify the health burden using hospitalization records or county-level incidence estimates, such as those from the Global Burden of Disease project [53], once more recent information becomes available. Nevertheless, our results are broadly consistent with epidemiological observations in New York State, demonstrating the capability and robustness of our modeling framework.

A notable finding is the scarcity of EPA AQS monitoring sites in most highly impacted counties, a significant challenge to tracking real-time air quality, issuing timely health advisories during extreme pollution events, and estimating the damage to public health in the aftermath, which could constrain public health responses, leave vulnerable populations at greater risk. Our modeling system helps fill this gap by identifying vulnerable populations and providing reliable health burden estimates. Expanding AQS monitoring networks to high-risk counties, particularly those identified by our modeling study, is critical for strengthening air quality management and protecting public health. Potential approaches include deploying mobile labs or integrating low-cost sensors into existing networks to help address gaps in current monitoring coverage. However, mobile labs cannot provide long-term monitoring, and low-cost sensors may exhibit large biases under extreme PM_2.5_ levels associated with wildfire events.

Finally, advancing simulation capabilities will be key to enabling rapid regulatory assessments. CAMS and QFED data are available within days after an event; leveraging such near real-time data could improve the timeliness and accuracy of air quality modeling and health risk evaluations. These advancements would provide reliable scientific evidence for health impact assessment and support exemptions for wildfire-related pollution events for air quality regulators at state and regional levels.

## Data Availability

All data that support the findings of this study are included within the article (and any supplementary files). The WRF model source code is available from the NCAR WRF repository (https://github.com/wrf-model/WRF). The CMAQ model can be accessed through the EPA Community Modeling and Analysis center (www.epa.gov/cmaq/access-cmaq-source-code). NCEP NFL products are provide by the NCAR Research Data Archive (https://rda.ucar.edu/datasets/d083002), and NAM forecast data are available at the NOAA National Centers for Environmental Information (www.ncei.noaa.gov/). NCAR WACCM products are distributed through www2.acom.ucar.edu/gcm/waccm. CAMS biomass burning emissions can be downloaded from Copernicus Atmospheric Data Store (https://ads.atmosphere.copernicus.eu/cdsapp#!/dataset/cams-global-fire-emissions-gfas). QFED products are accessible at https://portal.nccs.nasa.gov/datashare/iesa/aerosol/emissions/QFED/. Daily EPA AQS observations are accessible at www.epa.gov/outdoor-air-quality-data/download-daily-data.
